# Harnessing Filamentous Fungi for Enzyme Cocktail Production Through Rice Bran Bioprocessing

**DOI:** 10.3390/jof11020106

**Published:** 2025-01-31

**Authors:** Ana M. Yélamos, Jose F. Marcos, Paloma Manzanares, Sandra Garrigues

**Affiliations:** Food Biotechnology Department, Instituto de Agroquímica y Tecnología de Alimentos (IATA), Consejo Superior de Investigaciones Científicas (CSIC), Catedrático Agustín Escardino Benlloch 7, 46980, Valencia, Spain; ayelamos@iata.csic.es (A.M.Y.); jmarcos@iata.csic.es (J.F.M.); pmanz@iata.csic.es (P.M.)

**Keywords:** *Aspergillus*, *Penicillium*, cellulases, xylanases, bioactive peptides, antifungal proteins, food waste valorization, circular bio-economy

## Abstract

Valorization of agri-food residues has garnered significant interest for obtaining value-added compounds such as enzymes or bioactive molecules. Rice milling by-products, such as rice bran, have limited commercial value and may pose environmental challenges. Filamentous fungi are recognized for their ability to grow on residues and for their capacity to produce large amounts of metabolites and enzymes of industrial interest. Here, we used filamentous fungi to produce enzyme cocktails from rice bran, which, due to its polysaccharide composition, serves as an ideal substrate for the growth of fungi producing cellulases and xylanases. To this end, sixteen fungal strains were isolated from rice bran and identified at the species level. The species belonged to the genera *Aspergillus*, *Penicillium*, and *Mucor*. The *Aspergillus* species displayed the highest efficiency in cellulase and xylanase activities, especially *A. niger* var. *phoenicis* and *A. amstelodami*. *A. terreus*, *A. tritici*, and *A. montevidensis* stood out as xylanolytic isolates, while *P. parvofructum* exhibited good cellulase activity. *A. niger* var. *phoenicis* followed by *A. terreus* showed the highest specific enzymatic activities of α- and β-D-galactosidase, α-L-arabinofuranosidase, α- and β-D-glucosidase, and β-D-xylosidase. Additionally, proteomic analysis of *A. terreus*, *A. niger* var. *phoenicis*, and *P. parvofructum* exoproteomes revealed differences in enzyme production for rice bran degradation. *A. niger* var. *phoenicis* had the highest levels of xylanases and cellulases, while *P. parvofructum* excelled in proteases, starch-degrading enzymes, and antifungal proteins. Finally, two *Penicillium* isolates were notable as producers of up to three different antifungal proteins. Our results demonstrate that filamentous fungi can effectively valorize rice bran by producing enzyme cocktails of industrial interest, along with bioactive peptides, in a cost-efficient manner, aligning with the circular bio-economy framework.

## 1. Introduction

Social concerns aimed at reducing environmental pollution have driven Europe to transition towards a biologically based (bio-based) economy in search of environmentally friendly technologies and practices that can be used in the production of many industrial commodities. However, this new paradigm of sustainable development remains a challenge since new biocatalytic processes must compete with the already well-established and economically viable, but less eco-friendly, chemical processes [1,2].

Agri-food waste valorization has emerged as a prominent area within industrial biotechnology. Recently, there has been a great interest in the exploitation of these wastes as low-cost raw materials for the sustainable production of value-added compounds with application in many industries, such as the production of pulp and paper, food and feed, detergents, textiles, biofuels, and biochemicals [3]. These plant-based residues are of particular interest since they are an important reservoir of organic carbon. They mainly consist of polysaccharides (cellulose, hemicellulose, and pectin), the aromatic polymer lignin, proteins, and storage polysaccharides (e.g., starch, inulin, and gums), with their relative amount and structure varying depending on plant species [4]. However, one of the main obstacles to the use of this raw material is its recalcitrance due to the organization of its constitutive polymers [5].

Rice (*Oryzae sativa*) is the second most widely grown food crop after wheat. The rice processing industry produces a large quantity of by-products, which mainly include rice bran [6]. In 2022, a total of 726 million metric tons of unprocessed paddy rice was harvested, which generated 72.6 million metric tons of rice bran [7]. Rice bran, the brown outer layer of the rice kernel, is underutilized, often used as animal feed or discarded as waste. However, it is a nutrient-rich by-product, as it contains approximately 50% carbohydrates (mainly cellulose, hemicellulose –xylan–, and starch), 20% fat, 15% protein, 15% dietary fiber, and substantial amounts of vitamins and minerals [8]. Therefore, its complex composition makes it an ideal candidate for microbial degradation processes aimed at producing valuable enzymes, particularly cellulases and xylanases, which have a wide range of industrial applications [3]. Currently, the efficiency of commercial cellulases and hemicellulases for plant biomass processing, along with the associated costs, limit their utilization [9]. Therefore, to address these challenges, it is imperative to identify microbial strains that exhibit significant levels of highly active cellulases and hemicellulases (especially xylanases), and to uncover the conditions that best induce their bioproduction.

Filamentous fungi are eukaryotic organisms renowned for their ability to grow on diverse and economically viable substrates and for their ability to produce large amounts of metabolites, organic compounds, proteins, and enzymes [10], including those degrading complex plant polymers, known as carbohydrate active enzymes (CAZymes) (http://www.cazy.org/). Therefore, due to their extensive enzymatic repertoire, filamentous fungi are excellent candidates for the bioprocessing of agro-industrial by-products, such as rice bran, to produce cellulase- and xylanase-rich enzyme cocktails of industrial value.

In this study, we conducted a bioprospecting and identification of the mycobiota present in rice bran and explored the potential of 16 of the filamentous fungal isolates identified to degrade this by-product. We identified the most efficient cellulolytic and xylanolytic fungal strains and characterized their enzymatic profiles and exoproteomes, thus contributing to providing a sustainable and cost-effective method for cellulase and xylanase production, as well as other bioactive molecules, within the framework of a circular bio-economy.

## 2. Materials and Methods

### 2.1. Fungal Isolation and Growth Conditions

Fungal isolates (Table 1) were obtained from ground rice bran (DACSA). Rice bran was (i) incorporated onto the surface of potato dextrose agar (PDA, Difco, Leeuwarden, The Netherlands) plates (1 g/plate); or (ii) added to 50 mL of 10% potato dextrose broth (PDB, Difco) to a final concentration of 1%. In the case of the solid medium, rice bran-containing PDA plates were incubated for 7 days at 25 °C in the dark. The emerging fungal isolates were transferred to separate PDA plates until monosporic cultures were obtained. For the liquid medium, Erlenmeyer flasks were incubated for 48 h at 25 °C and 200 rpm. From each flask, 10, 25, and 50 µL of the medium were plated on PDA plates containing 50 µg/mL of chloramphenicol to prevent bacterial growth. This procedure was performed at 0, 24, and 48 h in triplicate. The plates were incubated for 5–7 days at 25 °C in the dark. Finally, fungal colonies were isolated and transferred to PDA plates to obtain monosporic cultures.

Growth profiles of the selected fungal strains were performed using *Aspergillus* Minimal Medium (AMM) [11] containing 25 mM of the monosaccharides D-glucose (Applichem GmbH, Darmstadt, Germany), D-xylose, L-arabinose, the disaccharide maltose (Sigma-Aldrich, St. Louis, MO, USA), or 1% cellulose (Merk Millipore, Burlington, MA, USA), birch wood xylan, soluble starch (Sigma-Aldrich), or rice bran. Strains were inoculated for up to 10 days at 25 °C.

### 2.2. Phenotypic Characterization of the Fungal Strains

The fungal isolates obtained from rice bran were phenotypically characterized through morphological observations. Isolates were three-point inoculated in duplicates with 5 µL of a suspension containing 10^5^ conidia/mL on four standardized solid culture media: PDA, malt extract agar (MEA), yeast extract agar (YES), and Czapek yeast extract agar (CYA) [12]. Plates were incubated at 25 °C in the dark for 7 days. Fungal isolates that showed similar characteristics were considered the same group. Selected fungal isolates that showed differential morphologies were identified at the species level by sequence analysis.

### 2.3. Molecular Identification of the Fungal Strains

Genomic DNA extraction was performed using the gDNA Isolation Kit (Roche, Mannheim, Germany) according to the manufacturer’s instructions. Fungal DNA was quantified by NanoDrop (Thermo Fisher Scientific, Wilmington, DE, US). For molecular identification, polymerase chain reaction (PCR) was performed using primers specific for internal transcribed spacer (ITS), ß-tubulin, and calmodulin sequences (Integrated DNA Technologies IDT, San Diego, CA, USA, Supplementary Table S1). PCR reactions were conducted in a total volume of 25 µL, containing 50 ng of DNA, 2.5 µL of 10× Taq Buffer, 0.25 µL of 10 µM dNTPs, 0.75 µL of 50 mM MgCl_2_, 0.5 µL of each 10 µM primer, and 0.12 µL of 1U DNA polymerase (BIOTAQ DNA Polymerase, Bioline, London, UK). The reaction mixtures were incubated at 94 °C for 5 min, followed by 35 cycles of 1 min at 94 °C, 1 min at 52 °C, 1 min at 72 °C, and a final extension of 5 min at 72 °C. PCR products were visualized using 1% agarose gel electrophoresis, stained with Midori Green Advance (Cultek, Düren, Germany), and purified with the Wizard^®^ SV Gel and PCR Clean-Up System (Promega, Madison, WI, USA). Sequencing reactions were labelled using the BigDye Terminator v3.1 Cycle Sequencing Kit (Thermo Scientific, Waltham, MA, USA). The PCR products were sequenced at the Central Service for Experimental Research (SCIE) at the University of Valencia (Spain). The resulting chromatograms were analyzed using the SNAPGene viewer (https://www.snapgene.com/snapgene-viewer). The obtained sequences were compared using BLASTn analysis (https://blast.ncbi.nlm.nih.gov/) with genomic sequences available in the public GenBank database at NCBI (https://www.ncbi.nlm.nih.gov/genbank/).

### 2.4. Phylogenetic Analysis

The nucleotide sequences from the rice bran isolated strains obtained from Sanger sequencing, along with the most similar sequences (best hits) identified through BLAST analysis, were aligned using the ClustalW algorithm [13] within the MEGA-7 software (https://www.megasoftware.net/). Either ITS, ß-tubulin, and/or calmodulin sequences of the reference strains *Aspergillus tritici* (Genbank sequence ID KC923428.1); *Aspergillus tamarii* (MT340979.1); *Aspergillus flavus* (MH279408.1); *Aspergillus chevalieri* (MN968349.1 and MZ027912); *Aspergillus niger* (OR511675.1); *Aspergillus amstelodami* (MK267406.1, KU569229.1, and HE974440); *Aspergillus montevidensis* (PP333898, MZ826409, and LC494260.1); *Aspergillus terreus* (MG576115.1); *Aspergillus tubingensis* (MK450659.1); *Penicillium parvofructum* (LT558882.1, (LT558999, and LT627646.1); *Penicillium italicum* (MT872095.1); *Penicillium capsulatum* (KF706674.1); *Penicillium roseopurpureum* (MW269249.1); *Penicillium chrysogenum* (KJ775613.1, KY469107, and MT210455.1); and *Mucor circinelloides* (OR816104.1 and KT207681.1) were obtained from the GenBank database. Sequence alignments were further refined by hand to ensure accuracy by checking misaligned regions, presence of gaps, ensuring conserved regions are aligned consistently, assessing alignment lengths, etc. These alignments were used to construct a phylogenetic tree using the maximum likelihood algorithm [14], given the presence of different fungal genera, including the distant genus *M. circinelloides*, which also served as the outgroup. The statistical significance of the resulting nodes was evaluated using the bootstrap test with 1000 pseudoreplicates [15].

### 2.5. Protein Production, Quantification, and SDS-PAGE Analyses

For protein production, 10^6^ conidia/mL of the chosen fungal isolates were inoculated in 250 mL Erlenmeyer flasks containing 50 mL of AMM supplemented with 2% rice bran as the sole carbon source. As the control of non-inducing enzyme production conditions, 10^6^ conidia/mL was inoculated in 50 mL of AMM supplemented with 2% D-fructose (Sigma-Aldrich). Cultures were inoculated in duplicates and were incubated at 25 °C for 7 days at 150 rpm. One mL supernatant aliquots were obtained after 1, 2, 3, 4, and 7 days of growth, and were subsequently centrifuged (5 min, 2400× *g*) and frozen (−20 °C) until further use.

The total protein present in the culture supernatants was measured at 4 days of growth in 96-well plates (Nunc) using the Bicinchoninic acid colorimetric assay (BCA, Sigma-Aldrich) following the manufacturer’s instructions.

SDS-PAGE analyses were performed in 12% polyacrylamide gels (40% Acrylamide/Bis solution, BIO-RAD, Hercules, CA, USA) [16]. A total of 10 µL of each supernatant sample was loaded per well. Proteins were visualized by Coomassie blue staining (Coomassie Brillian Blue, BIO-RAD). The experiments were repeated at least twice.

### 2.6. Enzymatic Activities

The cellulase and xylanase activities present in the 4-day-old supernatants were calculated using the colorimetric dinitrosalicylic acid (DNS) method (Sigma-Aldrich), which quantifies the release of reducing sugars. The assays were performed as indicated by [17]. Substrate solutions were prepared with 0.5% cellulose or 1% xylan in 0.02 M sodium phosphate buffer at pH 6.5. A total of 20 µL of each supernatant (in duplicate) was mixed with 20 µL of each substrate solution. For negative controls, 20 µL of the sodium phosphate buffer was mixed with 20 µL of each substrate solution. For the calibration curve, D-glucose and D-xylose standards were prepared from a stock solution of 4.5 mg/mL or 3.75 mg/mL, respectively, in the same buffer. The final concentrations for the calibration curve were obtained by using between 0 and 40 µL of the sugar stock solutions. Assays were performed in 96-well plates incubated at 45 °C, for 0.5 or 5 h for xylanase and cellulase activities, respectively. After incubation, 160 µL of DNS solution was added per well and a second incubation of 20 min was performed at 100 °C. Absorbance was measured by a spectrophotometer at a 570 nm wavelength. The enzymatic activity was expressed in µg of glucose/xylose equivalents per hour of incubation at 45 °C and pH 6.5.

The colorimetric *p*-4-nitrophenol (pNP) assays were performed using supernatant samples collected after 4 days of incubation in 96-well plates. The assays were performed as reported in [18], with minor modifications. Briefly, 10 μL of supernatant was mixed with 10 μL of 0.1% 4-nitrophenyl β-D-glucopyranoside for β-1,4-glucosidase (BGL) activity, 0.1% 4-nitrophenyl β-D-xylopyranoside for β-1,4-xylosidase (BXL) activity, 0.1% 4-nitrophenyl α-L-arabinofuranoside for α-L-arabinofuranosidase (ABF) activity, 0.1% 4-nitrophenyl α-D-galactopyranoside for α-1,4-galactosidase (AGL) activity, 0.1% 4-nitrophenyl β-D-galactopyranoside for β-1,4-galactosidase (LAC) activity, or 0.1% 4-nitrophenyl α-D-glucopyranoside for α-glucosidase (AGD) activity, 50 μL of 50 mM sodium acetate buffer (pH 5), and 30 μL of demineralized water in a final volume of 100 μL per well. All enzymatic activities were incubated for 30 min at 40 °C. The reaction was stopped by adding 100 µL of 0.25 M Na_2_CO_3_. The absorption values of technical triplicates were measured at a 405 nm wavelength. Assays were repeated at least twice. The enzymatic activity was expressed in mmol pNP/min/mL of supernatant.

### 2.7. Proteomics Analysis

Protein identification of the ~6 kDa Comassie stained band from *P. chrysogenum* RB10 SDS-PAGE gel was performed in the proteomics facility of the Spanish National Biotechnology Center (CNB, Madrid). The sample was loaded into an S-Trap™ column right after denaturation with 5% SDS, reduction and alkylation with tris (2-carboxyethyl) phosphine and chloroacetamide, and trypsin digestion [19]. Each digest was cleaned with a C18 StageTip prior to analysis by Liquid Chromatography Electrospray Ionization Tandem Mass Spectrometric (LC–ESI–MS/MS) analysis performed with an Ultimate 3000 nano HPLC system (Thermo Fisher Scientific, Waltham, MA, USA) coupled online to a Orbitrap Exploris 240 mass spectrometer (Thermo Fisher Scientific). For peptide quantification, 500 ng of each digest was injected in a total of 5 µL injection volume. Peptides were separated based on their polarity using a 50 cm ×  75 μm Easy-spray PepMap C18 analytical column. The raw data were exported, and a database search was conducted using Mascot Server v2.8.0 (Matrix Science, Chicago, IL, USA) and a target/decoy database built from sequences in the *P. chrysogenum* proteome at Uniprot Knowledgebase.

Proteins present in the low molecular weight bands from *P. parvofructum* AM8 and *A. niger* RT3 were identified in the proteomics facility of SCSIE University of Valencia. The gel slides were digested with trypsin as described [19]. A total of 20 µL of the digests was loaded in an Evotip pure tip (EvoSep, Odense, Denmark) according to the manufacturer instructions. LC–MS/MS was performed in a Tims TOF fleX mass spectrometer (Bruker, Billerica, MA, USA). The sample loaded in the Evotip pure tip was eluted to an analytical column (Endurance 8 cm × 100 µm, 3 µm; Evosep) by the Evosep One system, and resolved with the 100 SPD chromatographic method defined by the manufacturer. The eluted peptides were ionized in a captive Spray and analyzed in the ddaPASEF mode. The PASER system (Bruker) was used to search the MS and MSMS data with the Sequest algorithm (ProLuCID). The raw data obtained from *P. parvofructum* AM8 and *A. niger* RT3 were exported, and a database search was conducted against the Swiss-Prot database. In addition, MSFragger searches were performed (via FragPipe) for identification in the Uniprot *Aspergillus niger*_230621 database.

Protein identification from 4-day-old rice bran-containing supernatants of *A. terreus* AM39, *A. niger* var. *phoenicis* RT3, and *P. parvofructum* AM8 was performed in the proteomics facility of SCSIE University of Valencia. Briefly, proteins were precipitated with 10% trichloroacetic acid, the pellet was washed with acetone, and then resuspended in 100 µL of 50 mM ammonium bicarbonate. The correspondent volume to 10 µg of protein of every sample was taken, reduced, and alkylated. Digestion was subsequently performed with 400 ng of trypsin and stopped with 10% trifluoroacetic acid at a final concentration of 1%. A total of 1.5 µL of digested peptides were brought to 20 µL with 0.1% fluoroacetic acid and loaded in an Evotip pure tip (EvoSep) according to the manufacturer instructions. LC–MS/MS was performed in a Tims TOF fleX mass spectrometer. The samples loaded in the Evotip pure tip were eluted to an analytical column (EvoSep 15 cm × 150 µm, 1.5 µm; Evosep) by the Evosep One system, and resolved with the 30 SPD chromatographic method defined by the manufacturer. The eluted peptides were ionized in a captive Spray with 1700 V at 200 °C, and analyzed in the ddaPASEF mode. The system sensitivity was controlled with 50 ng of HELA digested proteins. The PASER system was used to search the MS and MSMS data with the ProLuCID. In addition, MSFragger searches were performed (via FragPipe) for the identification of the peptides.

In all cases, for protein identification, at least two peptides were considered, and the false discovery rate (FDR) was set to 1%. Protein abundance is represented by the ‘normalized spectral abundance factor (NSAF)’, which provides an improved measure for relative abundance by factoring the length of the protein into subsequent calculations. A NSAF value for a given protein is calculated by dividing the spectral counts (SpC) for a protein by its length (L). This value is then normalized by dividing by the sum of all SpC/L for all proteins identified in a complex mixture. NSAF values provide a measure of relative abundance and the ability to compare the abundance of proteins within a sample.

### 2.8. Identification of Antifungal Protein- (AFP) Coding Sequences

A total of 50 ng of *P. parvofructum* AM8 genomic DNA was used as a template for PCR amplification of the three genome-encoded AFPs using BIOTAQ DNA Polymerase with specific primers (Supplementary Table S1). PCR products were visualized using 1% agarose gel electrophoresis stained with Midori Green Advance and purified with the Wizard^®^ SV Gel and PCR Clean-Up System. Sequencing reactions were labelled using the BigDye Terminator v3.1 Cycle Sequencing Kit. The PCR products were sequenced at the SCIE (University of Valencia). Sequencing results were visualized, trimmed, and assembled using SeqMan Ultra^TM^, included in the DNAstar software (https://www.dnastar.com/), and sequence alignments were performed using the ClustalW algorithm.

### 2.9. Statistical Analysis

Data were analyzed by analysis of variance (ANOVA, LSD post-hoc test) using the Statgraphics Centurion XVIII (https://www.statgraphics.com/) software. The statistical significance was established at *p* < 0.05.

## 3. Results

### 3.1. Fungal Isolation from Rice Bran and Species Identification

After the isolation of fungal strains from both rice bran-containing liquid and solid media, a total of 28 filamentous fungal isolates were obtained, which were preliminarily characterized at the genera level based on morphological and cultural comparisons in four different media (PDA, MEA, CYA, and YES) [20]. Of these 28 isolates, 16 showed different phenotypes on both sides of the plates, such as mycelium texture, conidia color, morphology, and colony size (Supplementary Figure S1). A total of 10 fungal isolates were initially ascribed to the genus *Aspergillus* (AM3, AM9, AM13, AM15, AM39, RT3, RB13.2, RB13, RB9, and RB5.4), 5 to the genus *Penicillium* (AM29, AM8, AM27, RB10, and RT1) and 1 to the genus *Mucor* (RT4). These isolates were selected for further molecular identification at the species level using ITS/ β-tubulin/calmodulin sequence analyses. The sequencing results confirmed our morphological results, with *Aspergillus* being the predominant mycobiota present in rice bran. The isolates identified at species level are shown in Table 1. It is to be noted that in the case of RT3, sequencing results identified this strain at the subspecies level as *A. niger* var. *phoenicis*.

**Figure 1:**
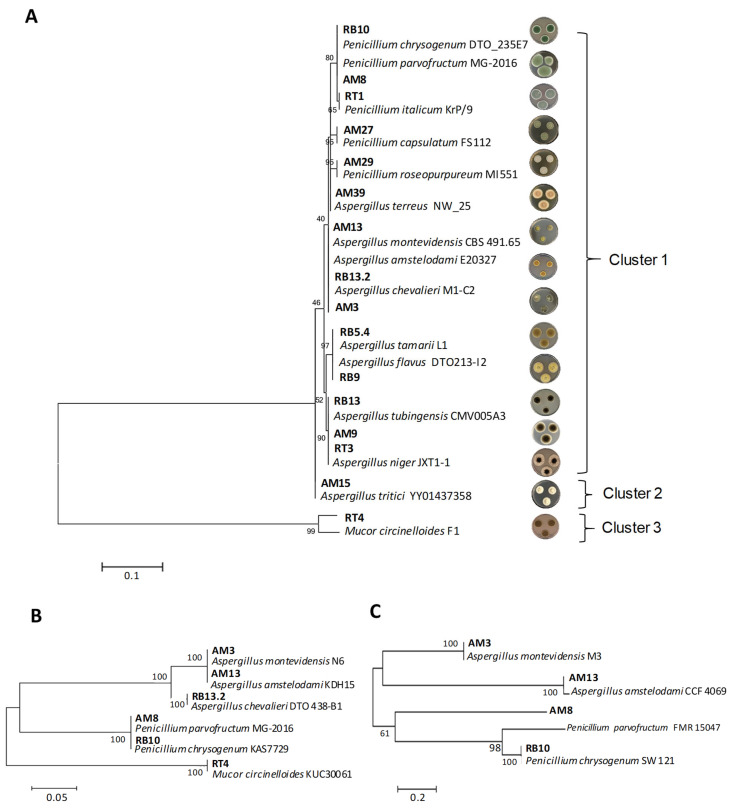
Maximum likelihood phylogenetic trees of rice bran-isolated fungi (in bold) constructed from the datasets of ITS (**A**), β-tubulin (**B**), and calmodulin (**C**) sequences. Codes are detailed in Table 1. The numbers on the nodes represent the frequency (%) with which a cluster appears in a bootstrap test of 1000 runs. Bootstrap values ≥40 are shown.

Phylogenetic analyses grouped the 16 identified species into three main clusters (Figure 1). For ITS sequences (Figure 1A), cluster 1 grouped most of the fungi under study, and included eight subclusters. Subcluster 1.1, which included both *P. parvofructum* AM8 and *P. chrysogenum* RB10 strains, is close to subcluster 1.2, which includes the strain of *P. italicum* RT1. Subclusters 1.3 and 1.4 included *P. capsulatum* AM27 and *P. roseopurpureum* AM29, respectively, which are distantly related from subcluster 1.5, grouping *A. terreus* AM39. Subcluster 1.6 groups the closely related species *A. montevidensis* AM3, *A. amstelodami* AM13, and *A. chevalieri* RB13.2. Subcluster 1.7 included the species *A. flavus* RB9 and *A. tamarii* RB5.4. Finally, subcluster 1.8 included both *A. niger* species (AM9 and RT3) and *A. tubingensis* RB13. Cluster 2 included *A. tritici* AM15, whereas cluster 3 included *M. circinelloides* AM15*,* which served as the outgroup.

It is of note that ITS sequences from *P. parvofructum* AM8 and *P. chrysogenum* RB10 (subcluster 1.1) and from *A. montevidensis* AM3, *A. amstelodami* AM13, and *A. chevalieri* RB13.2 (subcluster 1.6) were not informative enough to distinguish among closely related strains, and therefore, both β-tubulin and calmodulin sequences were also analyzed for these strains (Figure 1B,C). While β-tubulin sequences allowed the proper identification and phylogenetic separation of *A. chevalieri* RB13.2 from *A. amstelodami* AM13 and *A. montevidensis* AM3 (Figure 1B), calmodulin sequences allowed the identification and phylogenetic separation of *A. amstelodami* AM13 from *A. montevidensis* AM3, and of *P. parvofructum* AM8 from *P. chrysogenum* RB10 (Figure 1C).

### 3.2. Protein Secretion by Different Fungal Isolates

Once the fungal isolates were identified, a total of 14 strains were grown in rice bran-containing medium, and protein secretion patterns were compared (Supplementary Figure S2). The carbon source D-fructose, which shows low carbon catabolite repression-mediated gene repression [21], was used as a low/non-inducing control of enzyme production [22]. Additionally, *A. flavus* and *A. tamarii* were excluded from subsequent analyses due to their potential mycotoxigenic nature and their less competitive enzyme production abilities compared to other *Aspergillus* species [23,24]. In contrast, *A. terreus* was retained in the analyses due to its well-known enzyme production capabilities and industrial potential, despite the fact that some specific strains can produce secondary metabolites with toxic properties [25,26].

All isolates showed a good growth rate in both rice bran- and D-fructose-containing media, with higher growth observed in the former. SDS-PAGE analyses revealed a clear difference in protein production patterns when using rice bran or D-fructose as the sole carbon source, with overall protein production being muchnot sho higher in rice bran, as expected (Supplementary Figure S2). Among the species belonging to the genus *Aspergillus* (Supplementary Figure S2A–H), those that secreted the highest amounts of proteins into the rice bran-containing medium were *A. montevidensis*, *A. amstelodami*, and *A. tritici*. Regarding the genus *Penicillium* (Supplementary Figure S2I–M), *P. parvofructum* and *P. chrysogenum* were the highest protein producers. Finally, *M. circinelloides* secreted few proteins into the growth supernatant (Supplementary Figure S2N).

Despite the fact that virtually no proteins were secreted in the D-fructose-containing medium, in some specific cases, such as *P. chrysogenum, P. parvofructum*, and *A. niger* var. *phoenicis,* low molecular weight bands were clearly defined after 7 days of growth, which caught our attention (Supplementary Figure S2, red rectangles) and were sent for proteomic identification (Supplementary Data S1–S3). The results for *A. niger* var. *phoenicis* may indicate cell lysis, as proteomics analysis revealed that this band exhibited significant homology to the elongation factor 2, an essential intracellular protein catalyzing ribosomal translocation during protein synthesis [27] (Supplementary Data S1). In contrast, the results for the *P. chrysogenum* and *P. parvofructum* strains revealed protein homology primarily to the secreted protein PAFB [28] (Supplementary Data S2 and S3). PAFB belongs to the family of antifungal proteins (AFPs) produced by filamentous fungi. AFPs are small, cationic, cysteine-rich proteins that effectively inhibit the growth of human and plant pathogenic fungi at micromolar concentrations without causing cytotoxic effects on mammalian cells [29,30]. These proteins have been proposed as potential biofungicides and novel antifungal therapies.

In general, protein production in rice bran-containing medium showed the highest intensity after 4 and 7 days of growth, although in some cases, at day 7, protein decrease and/or degradation started to appear, as is the case, for example, of *A. montevidensis, A. amstelodami*, and *A. chevalieri* (Supplementary Figure S2A,C,G). Therefore, protein quantification was accounted at day 4 for all the strains under study (Table 2). Overall, *Penicillium* and *Aspergillus* species secreted a similar amount of proteins. For the *Penicillium* genus, protein amounts ranged from 0.99 mg/mL produced by *P. capsulatum* to 1.64 mg/mL by *P. parvofructum*. In the case of the *Aspergillus* genus, protein amounts ranged from 0.93 mg/mL produced by *A. montevidensis* to 2.17 mg/mL produced by *A. niger* var. *phoenicis*, the latter being the strain that produced the highest amount of proteins among all the isolates analyzed, which correlates with the results observed in the SDS-PAGE analysis (Supplementary Figure S2F). Finally, *M. circinelloides* showed significantly lower values of secreted proteins (0.43 mg/mL), as also depicted in Supplementary Figure S2N.

### 3.3. Assessment of Rice Bran Degradation Abilities Through Enzymatic Activity Determination in Different Fungal Isolates

**Figure 2:**
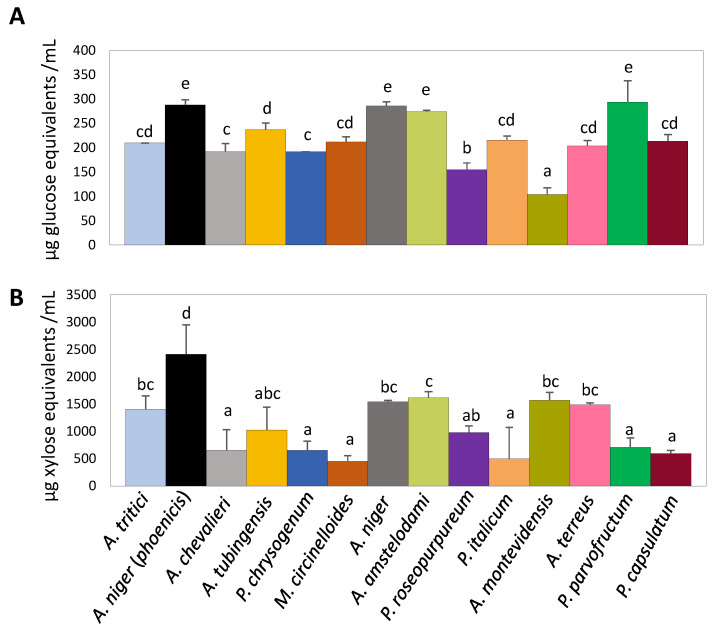
Determination of cellulase (**A**) and xylanase (**B**) activities present in rice bran-containing culture supernatants for the different fungal strains after 4 days of growth. Cellulase activity was expressed as μg of glucose equivalents/mL of supernatant after 5 h of incubation. Xylanase activity was expressed as μg of xylose equivalents/mL of supernatant after 30 min of incubation. Samples showing different letters show significant differences among the strains within each specific enzyme assay (ANOVA, LSD *p* < 0.05).

We determined and quantified both cellulase and xylanase activities present in the rice bran-derived culture supernatants after 4 days of growth of the different fungal isolates using the DNS method (Figure 2).

Cellulase activity ranged from 103.9 to 293.4 µg glucose equivalents/mL of culture supernatant (Figure 2A). Statistical analysis (ANOVA, *p* < 0.05) revealed that the two strains of *A. niger*, along with *A. amstelodami* and *P. parvofructum,* were the best producers of cellulolytic activity. These were followed by a large group that included *A. tritici*, *A. chevalieri*, *A. tubingensis*, *A. terreus*, *P. italicum*, *P. capsulatum*, *P. chrysogenum*, and *M. circinelloides*. The two fungi with the lowest enzymatic activity were *P. roseopurpureum* and *A. montevidensis*.

On the other hand, statistical analysis highlighted the high xylanase production capacity of *A. niger* var. *phoenicis* (2410.8 μg of xylose equivalents/mL of supernatant) (Figure 2B). The fungi with the lowest xylanase production capacity were *P. roseopurpureum*, *A. chevalieri*, *P. chrysogenum*, *M. circinelloides*, *P. italicum*, *P. parvofructum*, and *P. capsulatum*. These isolates showed statistically similar values, producing an average of ∼600 μg of xylose equivalents/mL of supernatant, except for *P. roseopurpureum*, which produced around 900 μg/mL. An intermediate group included *A. niger*, *A. amstelodami*, *A. terreus*, *A. tritici*, and *A. montevidensis*, which released approximately 1600 μg of xylose equivalents/mL of supernatant. It should be noted that the incubation period for determining xylanase activity (30 min) was significantly shorter than that used for cellulase activity determination (5 h), but the amount of reducing sugars released from xylan was greater than from cellulose, indicating that the fungi under study are preferentially xylanolytic in the conditions tested.

**Figure 3:**
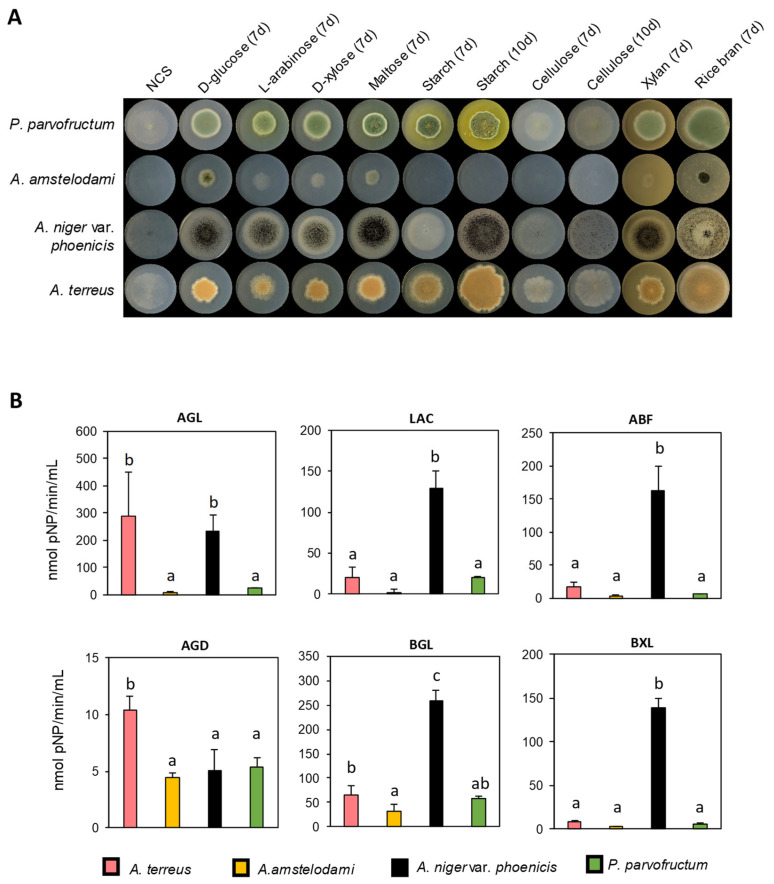
Evaluation of the degradation potential of *A. terreus* AM39, *A. niger* var. *phoenicis* RT3*, A. amstelodami* AM13, and *P. parvofructum* AM8. (**A**) Growth profiles on rice bran and rice bran-derived mono- and polysaccharides. NCS: no carbon source control. (**B**) Enzyme activity assays of the 4-day-old culture supernatants of rice bran-containing medium. Data represent mean values of biological duplicates and technical triplicates and the standard deviation (SD). AGL: α-1,4-D-galactosidase, LAC: β-1,4-D-galactosidase, ABF: α-L-arabinofuranosidase, AGD: α-glucosidase, BGL: β-1,4-D-glucosidase, BXL: β-1,4-xylosidase activity. Strains showing different letters indicate significant differences in enzymatic activity, while strains sharing the same letters show no significant difference within each specific assay, as determined by ANOVA LSD (*p* < 0.05). Note that statistical analyses cannot be compared among different enzymatic assays.

With these different enzymatic profiles, four fungal isolates (*A. niger* var. *phoenicis, A. amstelodami*, *P. parvofructum,* and *A. terreus*) were chosen for further characterization of growth capabilities (Figure 3A) and specific enzymatic activities required for rice bran degradation using the colorimetric pNP assays (Figure 3B). Among the chosen strains, *A. niger* var. *phoenicis, A. amstelodami*, and *P. parvofructum* stood out as the strains showing the highest cellulase activity (Figure 2). *A. niger* var. *phoenicis* also showed the highest xylanase activity, followed by *A. amstelodami.* Finally, *A. terreus* was chosen as a representative of a fungal strain with intermediate cellulase but high xylanase activity (Figure 2), for which we anticipated high potential for enzyme production based on previous reports [25].

The degradation abilities of these four selected isolates were assessed on solid minimal media containing rice bran and rice bran-related mono- and polysaccharides as carbon sources (Figure 3A). The no carbon source condition (NCS) was added as a control of residual growth. All isolates grew well on D-glucose, which was therefore used as another internal control to avoid misleading differences caused by general variations in growth speed. This condition already pointed *A. amstelodami* as the fungal isolate with the lowest growth rate among the four analyzed, and poor growth on most of the tested substrates was also observed for this strain. For the other three isolates, growth on the monosaccharides L-arabinose and D-xylose, the disaccharide maltose, and the polysaccharide xylan was similar to growth on D-glucose after 7 days of incubation, while strong differences were observed on starch and cellulose. While no growth on cellulose was observed for *A. amstelodami* at both time points analyzed (7 and 10 days), growth was observed for *P. parvofructum, A. niger* var. *phoenicis*, and *A. terreus* on pure cellulose, especially after 10 days of incubation. However, sporulation capacity was only observed for *A. niger* var. *phoenicis* at the latter time point. Finally, whereas the four chosen candidates showed strong ability to grow and sporulate on the complex substrate rice bran, *A. amstelodami* again stood out for its low growth rate, while *P. parvofructum* and *A. terreus* showed strong growth, even greater than that on D-glucose, highlighting their high potential for rice bran degradation and utilization. The growth of *A. niger* var*. phoenicis* on rice bran was also considerable, although its growth and sporulation capacity was different from that shown on D-glucose.

Among the specific enzymatic activities present in the 4-day-old supernatant of these four selected strains, we evaluated BGL, BXL, AGL, LAC, ABF, and AGD, which are key enzyme activities required for the degradation of the cellulose (BGL) and hemicellulose (BXL, AGL, LAC, and ABF) backbones of rice bran, and to a lesser extent, starch (AGD) (Figure 3B and Supplementary Data S4).

In general, *A. niger* var. *phoenicis* showed the best enzymatic activity performance among the four tested species, with the highest BXL (139.1 nmol pNP/min/mL), BGL (257.8 nmol pNP/min/mL), ABF (162.9 nmol pNP/min/mL), and LAC (128.6 nmol pNP/min/mL) activities, along with the second highest AGL activity (233.7 nmol pNP/min/mL), just after *A. terreus* (331.2 nmol pNP/min/mL), which correlates with its great growth potential on rice bran and rice bran-related sugars (Figure 3A). In contrast, *A. amstelodami* and *P. parvofructum* exhibited similarly low enzyme activity performance (Figure 3B and Supplementary Data S4), with *A. amstelodami* being the isolate showing the overall lowest enzymatic activity, which also correlated with the growth profile shown in Figure 3A. Therefore, these results together with the lower protein production levels of this strain in the culture supernatant (Table 2) led us to discard this isolate for further analysis. Finally, the starch-related AGD activity was in general low in the four tested species, with values ranging from 5 nmol pNP/min/mL in the case of *A. niger* var. *phoenicis* to 10.4 nmol pNP/min/mL in the case of *A. terreus.*

### 3.4. Proteomic Analyses

**Figure 4:**
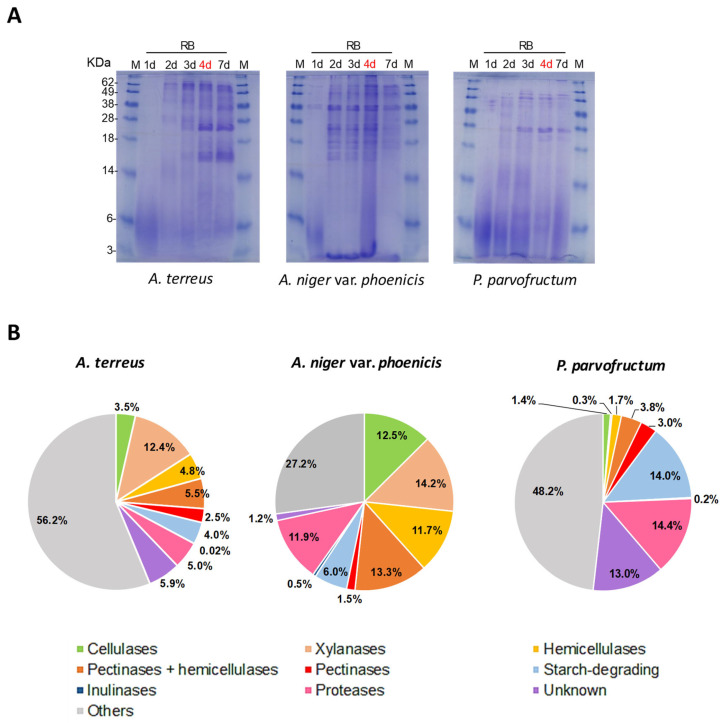
Analysis of the *A. terreus* AM39*, A. niger* var. *phoenicis* RT3, and *P. parvofructum* AM8 exoproteomes. (**A**) SDS-PAGE analyses of secretomes (3 × concentrated) after 1, 2, 3, 4, and 7 days (d) of growth in rice bran (RB)-containing medium. Day 4, which was the condition chosen for proteomics studies, is highlighted in red. (**B**) Distribution of extracellular proteins secreted in rice bran. Numbers in the diagrams represent the relative abundances (in percentages) of the proteins/enzymes detected in each category.

Proteomics studies were conducted to characterize the exoproteome of *A. terreus, A. niger* var. *phoenicis*, and *P. parvofructum* after 4 days of growth in rice bran-containing medium (Figure 4A, in red) (Supplementary Data S5–S7). The identified proteins were functionally classified according to their biological roles primarily related to plant biomass degradation (Figure 4B). For simplicity, we grouped the main enzymes based on activity targeting main polysaccharide components. The accessory enzymes that hydrolyze both pectin (side chain) and hemicellulose linkages (e.g., ferulolyl sterases, α-L-arabinofuranosidases, and β-galactosidases) were categorized as the ‘pectinases + hemicellulases’ group, whereas the enzymes that hydrolyze hemicelluloses (excluding xylan) but not pectin (e.g., fucosidases, mannanases, and mannosidases) were classified as the ‘hemicellulases’ group.

In total, 745 proteins were identified in the *A. terreus* secretome, while 382 and 263 proteins were identified in *A. niger* var. *phoenicis* and *P. parvofructum,* respectively. In all cases, hemicellulases were more abundant than cellulases, and pectinases and inulinases were overall underrepresented, reflecting the high hemicellulose content, particularly xylan, and low pectin and inulin content within the rice bran composition. Among the three isolates, *A. niger* var. *phoenicis* showed the highest amount of hemicellulases, including xylanases (39.2% of the secretome), followed by *A. terreus* (22.7%) and *P. parvofructum* (5.8%). These results correlate with the xylanase activity of these three isolates, with *A. niger* var. *phoenicis* displaying the highest xylanase activity, followed by *A. terreus* and *P. parvofructum* (Figure 2B).

*A. niger* var. *phoenicis* was, additionally, the isolate with the highest cellulase production (12.5%) followed by *A. terreus* (3.5%) and *P. parvofructum* (1.4%). However, these data do not correlate with the cellulase activity of these fungi, since both *A. niger* and *P. parvofructum* exhibited similar cellulase activities, followed by *A. terreus* (Figure 2A).

Regarding the putative starch-degrading capability of the three isolates, *P. parvofructum* stands out as the isolate with the highest abundance of starch-degrading enzymes induced in the presence of rice bran (14%), while *A. niger* var. *phoenicis* and *A. terreus* exhibited similar levels (6 and 4%, respectively). Similarly, the abundance of proteases/peptidases identified in the *P. parvofructum* secretome (14.4%) is higher than in the cases of *A. niger* var. *phoenicis* (11.9%) and *A. terreus* (5%).

Interestingly, while approximately 60% of the *A. niger* var. *phoenicis’* secretome consists of CAZymes, *A. terreus* and *P. parvofructum* CAZymes account for 33 and 23% of the secretome, respectively. These data confirm *A. niger* as one of the most efficient ascomycete filamentous fungi for plant biomass degradation. However, *A. terreus* and *P. parvofructum* are also promising candidates not only for enzyme cocktail production, but also for the production of other proteins and peptides with different bioactive properties from rice bran beyond CAZymes. For example, the exoproteome of *A. terreus* revealed the production of lipases (0.6%), chitinases (1.3%), catalases (2.3%), and uncharacterized cysteine-rich proteins and peptides (1.2%), including putative antifungal peptides, which could potentially have bioactive properties (Supplementary Data S5). Notably, *P. parvofructum* also revealed the production of small cysteine-containing peptides comprising a total of 4.6% of the secretome, which included two different proteins belonging to the AFP family: a class A AFP (protein ID A0A8H4WM31), comprising 2.1% of the secretome (Supplementary Data S7), and to a lesser extent, a class C AFP (ID A0A167U5W7), which accounts for 0.6%. This is in marked contrast with the production of a class B AFP by the same *P. parvofructum* in D-fructose-containing medium (Supplementary Data S2). Therefore, the results of the rice bran-induced exoproteome revealed the presence of class A and C AFP proteins produced by this fungus and indicated the absence of the class B AFP. Given the relevance of AFPs as antifungal agents, we decided to further characterize the protein sequences of the classes A, B, and C encoded in the *P. parvofructum* genome, which has not yet been publicly released. To this end, specific primers were designed (Supplementary Table S1) and PCR products obtained after the amplification of *P. parvofructum* genomic DNA were sequenced. The results revealed that the nucleotide sequences of *P. parvofructum* AFPs were almost identical to the well characterized class A PAF [31], class B PAFB [28], and class C PAFC [32] proteins from *P. chrysogenum*, with small nucleotide differences located either in the signal peptide or in the intronic sequences (Supplementary Figure S3), which do not influence the mature amino acid sequences of the corresponding AFPs. These results confirm the sequence similarity and thus phylogenetic closeness between *P. chrysogenum* and *P. parvofructum*, as depicted in Figure 1, and unveils the growth conditions for the production of each of the different AFPs encoded in *P. parvofructum*.

## 4. Discussion

Agri-food waste valorization has become a key focus in industrial biotechnology, driven by the growing interest in utilizing these abundant residues as renewable resources within the circular bio-economy framework. In this study, we focused on the valorization of rice bran, the primary by-product of rice milling, through its bioprocessing by different filamentous fungal species. Filamentous fungi are key players in plant biomass valorization due to their ability to produce a wide range of enzymes that can break down the complex polymers in lignocellulosic material [10], which offer a great advantage compared to alternative methods of waste valorization, such as chemical, mechanical, and even bacterial treatments. While chemical methods can efficiently break down lignocellulosic materials, they often involve harsh chemicals, generate toxic by-products, and have high energy requirements [33]. Mechanical methods (such as grinding and milling), though simple and cost-effective, require substantial energy and are less efficient in fully breaking down complex compounds such as lignin [34], resulting in lower valorization efficiency. Bacterial fermentation, although versatile, often lacks the wide enzymatic capacity of fungi [35], leading to less efficient waste conversion. In contrast, filamentous fungi produce a broad range of enzymes that specifically target complex plant polymers like cellulose and hemicellulose, as confirmed here for *A. niger* and shown for *A. terreus* and *P. parvofructum,* making them a more sustainable, eco-friendly, and cost-effective option for valorizing agri-food wastes such as rice bran. Additionally, they can produce bioactive compounds as part of their active metabolism. Therefore, fungal (bio)technology offers an environmentally friendly and low-cost solution for plant biomass conversion.

The filamentous fungal species used in this study were directly isolated from rice bran based on the hypothesis that fungi comprising the mycobiota present in the residue would theoretically ensure the production of high yields of the enzymes required for its degradation [36]. Accordingly, we isolated and identified ten species from the genus *Aspergillus,* six from the genus *Penicillium,* and one from the genus *Mucor,* as shown in Figure 1 and Supplementary Figure S1. These three most dominant genera in rice bran samples, with *Aspergillus* being the most prevalent genus, represent the highest overall frequencies of occurrence in agro-waste feedstocks, alongside other *Trichoderma* and *Fusarium* species, as reported in substrates such as rotten wood [37], rice straw, wheat straw, sugarcane bagasse [38], and castor bean waste [39]. Whereas *Aspergillus* and *Penicillium* have a long history of producing enzymes and secondary metabolites with industrial value [40,41,42], the less-studied *Mucor* genus is of significant interest due to the production of enzymes and bioactive compounds, for its role in fermentation processes, and its bioremediation potential [43,44,45]. Our rice bran-derived *M. circinelloides* RT4 strain showed intermediate cellulolytic enzymatic activity, but very low xylanolytic activity, and produced minimal protein amounts when grown in rice bran-containing medium compared to the other fungal isolates under study (Figure 2, Supplementary Figure S2, and Table 2). Therefore, these results, along with its unknown pathogenic potential for causing mucormycosis [46], led us to exclude it from further analysis. In contrast, *A. niger* strains, in particular *A. niger* var. *phoenicis* RT3, emerged as the best fungal candidate for rice bran bioprocessing in terms of growth and enzymatic potential. This strain showed the highest protein production among the isolates analyzed (Table 2), the highest cellulolytic and xylanolytic activities (Figure 2), good degradation and utilization potential of rice bran and related carbohydrates, including the recalcitrant cellulose (Figure 3A), and also exhibited the highest BGL-, BXL-, ABF-, and LAC-specific enzyme activities, sharing similar high AGL values to those of *A. terreus* AM39 (Figure 3B). These results correlate with previous studies in which *A. niger* exhibited high specific enzymatic activities when grown on alternative (hemi-)cellulose-rich by-products such as wheat bran [18]. Moreover, these results demonstrate the potential of *A. niger* var. *phoenicis* RT3 to produce enzyme cocktails that are critical for rice bran bioprocessing and have potential industrial applications. β-glucosidases are enzymes that hydrolyze glycosidic bonds in β-D-glucosides, releasing glucose from cellulose. Their role in converting complex carbohydrates into simpler sugars makes them particularly valuable in food and biofuel production [47]. β-D-xylosidases catalyze the hydrolysis of xylo-oligosaccharides into xylose. The versatility and efficiency of β-xylosidases make them critical in the conversion of plant biomass for biofuel production, environmentally friendly paper production, prebiotic formulation, and animal feed [48]. α-L-arabinofuranosidases hydrolyze arabinofuranosidic linkages in hemicelluloses and pectins. These enzymes are crucial for the breakdown of polysaccharides into fermentable sugars for bioethanol, and are also applied in the food industry for enhancing juice clarification and for animal feed [49]. β-D-galactosidases catalyze the hydrolysis of β-galactosidic bonds from hemicelluloses and pectins. They have various industrial applications particularly in dairy production and food processing due to their ability to break down lactose and other galactosides [50]. Finally, α-D-galactosidases hydrolyze α-galactosidic bonds in hemicelluloses, especially mannans and glucans, and are applied for enhancing digestibility and nutritional value in several food products, biofuel production, and probiotic formulation [51].

In addition to the enzymatic activities, the exoproteome of *A. niger* var. *phoenicis* was also analyzed after growth in rice bran. Approximately 40% of the secretome was composed of hemicellulases, including xylanases, which aligns with the hemicellulase composition of the exoproteome found in a different *A. niger* strain when grown in wheat bran-containing medium (~36%) [18]. Remarkably, the most abundant protein in the secretome of the *A. niger* var. *phoenicis* RT3 was the endo-1,4-β-xylanase XynB*,* accounting for almost 6% of the total secretome (Supplementary Data S6). XynB, a glycosil hydrolase (GH) 11 xylanase, has been reported to exhibit the best xylanase performance in xylan degradation among the five most highly induced endo-xylanases in *A. niger* (xynA, xynB xynC, XynD, and XynE/5) [52], further reinforcing the enormous potential of *A. niger* for plant biomass degradation and bioconversion [18].

On the other hand, *P. parvofructum* AM8 was the best producer of cellulolytic activity after *A. niger,* showing growth capability on cellulose (Figure 3A) and moderate BGL activity comparable to that of *A. terreus* (Figure 3B). However, the xylanolytic activity of this isolate was lower than other tested candidates (Figure 2) but enough to support growth on xylan as sole carbon source (Figure 3A). Despite the relatively low activity of the specific enzymatic activities analyzed (Figure 3B), *P. parvofructum* exhibited good potential for rice bran degradation and utilization, as depicted in Figure 3A, which highlights (i) the possible discrepancies that may exist in enzyme affinities between artificial pNP substrates and natural substrates during rice bran degradation, (ii) the necessity of a whole synergistic arrangement of enzymes for efficient plant biomass degradation, and (iii) possible discrepancies between enzyme activities present in liquid and solid media [53].

Even though strains of the genus *Penicillium* have not been as extensively exploited as those of the genera *Aspergillus* or *Trichoderma* for enzyme production [54,55], several studies have described *Penicillium* strains as good cellulolytic organisms, in some cases even surpassing *Trichoderma* in performance [56]. Therefore, *Penicillium* species are emerging as prominent players in cellulase production, especially in the conversion of biomass for biofuel applications. In the case of *P. parvofructum,* the cellulolytic activity present in the culture medium, which was comparable to that of *A. niger* strains (Figure 2), did not correlate with cellulase abundance in the exoproteome (Figure 4). In fact, only 1.4% of the exoproteome accounted for cellulases in this strain. This discrepancy may be explained by (i) the high efficiency of *P. parvofructum* cellulases, which opens up possibilities for novel enzyme characterization and enzyme cocktail generation, and/or (ii), the presence of additional cellulases in the secretome with no functional annotation, given the large amount of unknown proteins in *P. parvofructum* (13%) and the lack of genome sequencing available in public databases. In any case, the characterization of the cellulases produced by this poorly studied *Penicillium* species deserves further investigation and will be addressed in the future. Similarly, the fact that relatively low xylanase activity (Figure 2), combined with low xylanase abundance in the exoproteome (Figure 4B), still supports strong growth on xylan as the sole carbon source (Figure 3A) suggests high enzymatic efficiency of *P. parvofructum* xylanases, which will be further investigated in future studies.

Beyond the interest related to *P. parvofructum* cellulases and xylanases, this species also stands out for the production of starch-degrading/modifying enzymes and proteases, both accounting for approx. 14% of the exoproteome. Both enzyme classes play critical roles in making industrial processes more efficient, eco-friendly, and cost-effective, and have potential applications in food processing, detergents, pharmaceuticals, and paper and textile processing [57,58].

Among the secreted proteins identified for *P. parvofructum* (Figure 4) (Supplementary Data S3), antifungal AFPs deserve special attention due to their potential as antifungal agents in multiple fields [29,30]. Although the potent antifungal activity of several AFPs have been demonstrated both in vitro and in vivo [29,30,59,60,61], one of the major bottlenecks for AFP application at an industrial level is the identification of culture conditions triggering AFP secretion. In this regard, we have gone a step forward in the identification of two culture conditions that induce the secretion of the three different AFPs encoded in *P. parvofructum*, which are orthologous to the already described *P. chrysogenum* PAF [31], PAFB [28], and PAFC [32] (Supplementary Figure S3). It is not uncommon to find AFPs sharing the same amino acid sequences encoded in different but closely related species, as previously observed between *P. chrysogenum* and *Penicillium roqueforti,* and among different *Fusarium* species [62]. The conditions that trigger PAFB secretion in *P. chrysogenum* remain intriguing, since gene expression does not always correlate with protein production in the culture supernatant [28]. Interestingly, PAFB production by its native fungus has only been observed under nutrient-excess conditions, contrasting with most of the secreted AFPs, for which nutrient limitation and unfavorable growth conditions are typically the major triggers for their production [63]. In this study, we have demonstrated that alternative growth conditions consisting of minimal medium with D-fructose as the sole carbon source and at the late growth phase can also induce the production of this protein in both *P. chrysogenum* (Supplementary Data S2) and *P. parvofructum* (Supplementary Data S3). In addition to PAFB, *P. parvofructum* also produced PAF and PAFC orthologs when grown in minimal medium with rice bran as the sole carbon source, with PAF being more abundant (Supplementary Data S7). To the best of our knowledge, this is the first time that AFP production has been reported from a medium based on agricultural by-products, such as rice bran, paving the way for the sustainable, cost-efficient bioproduction of AFP cocktails for industrial applications.

Finally, another interesting candidate for rice bran valorization was the *A. terreus* AM39 strain. *A. terreus* is a well-known industrially relevant filamentous fungus, with a long history of use in the biotechnological production of organic acids, particularly itaconic acid, as well as cholesterol-lowering compounds like lovastatin [64,65]. Additionally, a genome-wide analysis of *Aspergillus* section *Terrei*, to which *A. terreus* belongs, has recently demonstrated the high potential of these species for secondary metabolite production and plant biomass degradation [25]. These facts, along with its already wide industrial acceptance, make *A. terreus* a strong candidate for rice bran valorization through the production of enzymatic cocktails and other bioactive compounds. In this study, the *A. terreus* AM39 strain produced the second-highest amount of extracellular proteins during growth in rice bran (Table 2), together with *P. parvofructum* and just after *A. niger* var. *phoenicis.* In addition, the rice bran-derived culture supernatants of the *A. terreus* AM39 strain exhibited high xylanolytic activity, just below *A. niger* var. *phoenicis* and comparable to that of *A. niger* AM9*,* and intermediate cellulolytic activity (Figure 2). Moreover, *A. terreus* showed strong growth on rice bran and all rice bran-related mono- and polysaccharides, including cellulose and starch (Figure 3A), and very high AGL-specific enzymatic activity, comparable to that of *A. niger* var. *phoenicis,* the second highest BGL activity, comparable to that of *P. parvofructum,* and the highest AGD activity (Figure 3B). These results positioned *A. terreus* as the second most interesting isolate, after *A. niger* var *phoenicis,* for rice bran valorization among the four final candidates, and therefore, the exoproteome of this species grown in rice bran-containing medium was investigated (Figure 4). This exoproteome was the most diverse from the three final candidates, with more than 700 proteins identified. Of these, 33% represent CAZymes involved in plant biomass degradation, while more than 56% comprise other proteins, such as lipases, proteases, catalases, chitinases, cutinases, effectors, small cysteine-rich proteins, and putative antifungal proteins different from canonical AFPs [62], among others. The majority of these proteins remain uncharacterized, making *A. terreus* AM39 an ideal candidate for further exploration in enzyme discovery and biotechnological applications, particularly in the development of novel enzyme cocktails for biomass valorization and industrial processes. However, despite the biotechnological potential of *A. terreus* AM39, we have not evaluated its ability for mycotoxin production in this study. Some *A. terreus* strains have been described to produce several toxic compounds, such as territrems, under specific growth conditions [66]. Even though mycotoxin production has been demonstrated to be both strain- and growth condition-dependent, if finally produced, mycotoxins can most likely be avoided by properly controlling the fermentation conditions [67]. Therefore, *A. terreus* still remains a valuable source for enzyme production, particularly in industrial applications where the benefits may outweigh the risks posed by potential toxin-producing strains. However, it is also worth noting that several *A. terreus* strains are emerging as opportunistic human pathogens, causing infections like aspergillosis in immunocompromised individuals [68]. This dual role highlights the need for careful management in industrial settings.

In this study, we evaluated the individual cellulolytic and xylanolytic potential of 14 fungal isolates and characterized the exoproteome of the promising *A. terreus*, *P. parvofructum*, and *A. niger* var. *phoenicis*. Although the latter appeared to be the best candidate for plant biomass degradation, we cannot dismiss the possibility of combining these isolates for their application as synthetic microbial consortia in industrial processes. Synthetic microbial consortia have emerged to recreate the natural microbial communities that coexist in the soil where lignocellulose degradation occurs, offering several advantages in terms of robustness and the potential for novel metabolic pathways [69]. This approach could lead to the production of complementary enzymes, particularly GHs, required for efficient plant biomass degradation [70]. Therefore, the evaluation of fungal synthetic consortia for rice bran valorization will be addressed in the future.

Additionally, with the recent advances in fungal synthetic biology [71,72,73] and genome editing [74,75], precision fermentation techniques can be applied to design optimized enzyme cocktails tailored for specific biomass types. For example, chimeric transcription factors can enhance/modify the expression of target enzymes in fungi, leading to more efficient and/or directed degradation processes, as demonstrated in *A. niger* [22], *Penicillium oxalicum* [76], and *T. reesei* [77]. This progress not only paves the way for more effective plant biomass valorization but also supports the development of sustainable biotechnological solutions in industrial applications. As we move forward, the integration of engineered fungi and synthetic microbial consortia will be crucial in unlocking new strategies for biomass conversion, ultimately fostering a more sustainable and eco-friendly industrial landscape.

## 5. Conclusions

In conclusion, this study highlights the ability of filamentous fungi to effectively valorize rice bran, a commonly underutilized agricultural residue, through the production of valuable enzyme cocktails and bioactive peptides. Our findings demonstrate that specific fungal isolates, particularly from the *Aspergillus* genus, are proficient in producing plant biomass-degrading enzymes that are crucial for many industrial applications, whereas *Penicillium* isolates are relevant for antifungal peptide production. The proteomic analysis provided additional insights into their enzyme profiles, further demonstrating their metabolic diversity. By adopting this approach within the circular bio-economy framework, we can mitigate the environmental issues associated with agricultural waste while enhancing the economic potential of these by-products, paving the way for sustainable biotechnological innovations.

## Figures and Tables

**Table 1:** Strains isolated from rice bran and characterized in this study.

Code	Species	GenBank Accession Number		
		ITS	ß-Tubulin	Calmodulin
AM3	*Aspergillus montevidensis*	PQ483919	PQ520635	PQ609700
RB9	*Aspergillus flavus*	PQ483907	-	-
RB5.4	*Aspergillus tamarii*	PQ483908	-	-
AM9	*Aspergillus niger*	PQ483905	-	-
AM13	*Aspergillus amstelodami*	PQ483911	PQ520634	PQ609696
AM15	*Aspergillus tritici*	PQ483909	-	-
AM39	*Aspergillus terreus*	PQ483910	-	-
RT3	*Aspergillus niger* var. *phoenicis*	PQ483906	-	-
RB13.2	*Aspergillus chevalieri*	PQ483912	PQ520633	-
RB13	*Aspergillus tubingensis*	PQ483904	-	-
AM29	*Penicillium roseopurpureum*	PQ483916	-	-
AM8	*Penicillium parvofructum*	PQ483914	PQ520632	PQ609699
AM27	*Penicillium capsulatum*	PQ483917	-	-
RB10	*Penicillium chrysogenum*	PQ483915	PQ520631	PQ609698
RT1	*Penicillium italicum*	PQ483913	-	-
RT4	*Mucor circinelloides*	PQ483918	PQ609697	-

-: not determined.

**Table 2:** Total protein produced by the different fungal species grown in a rice bran-containing medium after 4 days of growth. Distinct letters correspond to differences in statistical significance (ANOVA, *p* < 0.05).

Fungi	[Protein] (mg/mL)
*A. montevidensis* AM3	0.93 ± 0.01 ^b^
*A. niger* AM9	1.27 ± 0.09 ^bcde^
*A. amstelodami* AM13	1.38 ± 0.04 ^cde^
*A. tritici* AM15	1.30 ± 0.03 ^bcde^
*A. terreus* AM39	1.64 ± 0.08 ^e^
*A. niger* var. *phoenicis* RT3	2.17 ± 0.01 ^f^
*A. chevalieri* RB13.2	1.48 ± 0.11 ^de^
*A. tubingensis* RB13	1.27 ± 0.08 ^bcde^
*P. roseopurpureum* AM29	1.57 ± 0.73 ^de^
*P. parvofructum* AM8	1.64 ± 0.03 ^e^
*P. capsulatum* AM27	0.99 ± 0.06 ^bc^
*P. chrysogenum* RB10	1.29 ± 0.01 ^bcde^
*P. italicum* RT1	1.14 ± 0.09 ^bcd^
*M. circinelloides* RT4	0.43 ± 0.01 ^a^

## Data Availability

DNA sequences from each identified fungal isolate obtained through Sanger sequencing were deposited in the GenBank database, and accession numbers are shown in Table 1. All data generated or analyzed in this study are included in this published article and the public repository DIGITAL CSIC.

## Supplementary Materials

The following supporting information can be downloaded at: https://www.mdpi.com/article/10.3390/jof11020106/s1, Figure S1: Phenotypic characterization in CYA, MEA, PDA, and YES media of the different filamentous fungi isolated from rice bran.; Figure S2: SDS-PAGE analysis of the proteins secreted by fungal strains isolated from rice bran; Figure S3: Sequencing results and protein identification of *P. parvofructum* AFPs. Table S1: Primers used in this study. Supplementary Material Data S1–S7. Harnessing filamentous fungi for enzyme cocktail production through rice bran bioprocessing.
