# Supplementary material for: Harnessing Filamentous Fungi for Enzyme Cocktail Production Through Rice Bran Bioprocessing
Source: J Fungi (Basel). 2025 Jan 31;11(2):106. doi: 10.3390/jof11020106 (PMC11856480; doi:10.3390/jof11020106)
Supplement: Supplementary file 1 [file jof-11-00106-s001.zip › Supplementary Tables & Figures.pdf]

Supplementary Tables and Figures

Harnessing filamentous fungi for enzyme cocktail production through rice bran bioprocessing

Ana M. Yélamos, Jose F. Marcos, Paloma Manzanares and Sandra Garrigues*

Food Biotechnology Department, Instituto de Agroquímica y Tecnología de Alimentos (IATA), Consejo Superior de Investigaciones Científicas (CSIC), Catedrático Agustín Escardino Benlloch 7, 46980 Paterna, Valencia, Spain

* Correspondence: Sandra Garrigues: sgarrigues@iata.csic.es

Supplementary Table 1. Primers used in this study.

Locus	ID	Use*	Sequence (5´- 3´)	Tm (ºC)	References
Internal Transcribed Spacer (ITS)	ITS5	F	GGAAGTAAAAGTAAAAGTCGTAACAAGG	55	[1]
	ITS4	R	TCCTCCGCTTATTGATATGC	52	[2]
ß-tubulin	Bt2a	F	GGTAACCAAATCGGTGCTGCTTTC	58	[3]
	Bt2b	R	ACCCTCAGTGTAGTGACCCTTGGC	62	
calmodulin	CMD5	F	CCGAGTACAAGGARGCCTTC	56	[4]
	CF4	R	TTTYTGCATCATRAGYTGGAC	52	[5]
PparA	PAF_SPFw1	F	ATGCAAATCACCACAGTTGC	54	This study
	PAF_SPRv	R	CTAGTCACAATCGACAGCG	53	This study
PparB	PAFB_SPFw	F	ATGCAGATCACCAGCATTGC	56	This study
	PAFB_SPRv	R	TCAAACTGGGGTCTGGCAG	57	This study
PparC	PAFC_SPFw	F	ATGAAGGTTACTGCTCTCC	51	This study
	PAFC_SPRv	R	CTAGCATCTGGCTCCCCC	57	This study

*F: forward; R: reverse.

References

1. White, T. Amplification and direct sequencing of fungal ribosomal RNA genes for phylogenetics. *PCR Protocols: a guide to methods and applications/ Academic Press, Inc* **1990**.
2. Visagie, C.M.; Houbraken, J.; Frisvad, J.C.; Hong, S.B.; Klaassen, C.H.; Perrone, G.; Seifert, K.A.; Varga, J.; Yaguchi, T.; Samson, R.A. Identification and nomenclature of the genus *Penicillium*. *Stud Mycol* **2014**, *78*, 343-371, doi:10.1016/j.simyco.2014.09.001.
3. Glass, N.L.; Donaldson, G.C. Development of primer sets designed for use with the PCR to amplify conserved genes from filamentous ascomycetes. *Appl Environ Microbiol* **1995**, *61*, 1323-1330, doi:10.1128/aem.61.4.1323-1330.1995.
4. Hong, S.-B.; Cho, H.-S.; Shin, H.-D.; Frisvad, J.C.; Samson, R.A. Novel *Neosartorya* species isolated from soil in Korea. *Int J Syst Evol Microbiol* **2006**, *56*, 477-486, doi:https://doi.org/10.1099/ijs.0.63980-0.
5. Peterson, S.W.; Vega, F.E.; Posada, F.; Nagai, C. *Penicillium coffeae*, a new endophytic species isolated from a coffee plant and its phylogenetic relationship to *P. fellutanum*, *P. thiersii* and *P. brocae* based on parsimony analysis of multilocus DNA sequences. *Mycologia* **2005**, *97*, 659-666, doi:10.3852/mycologia.97.3.659.

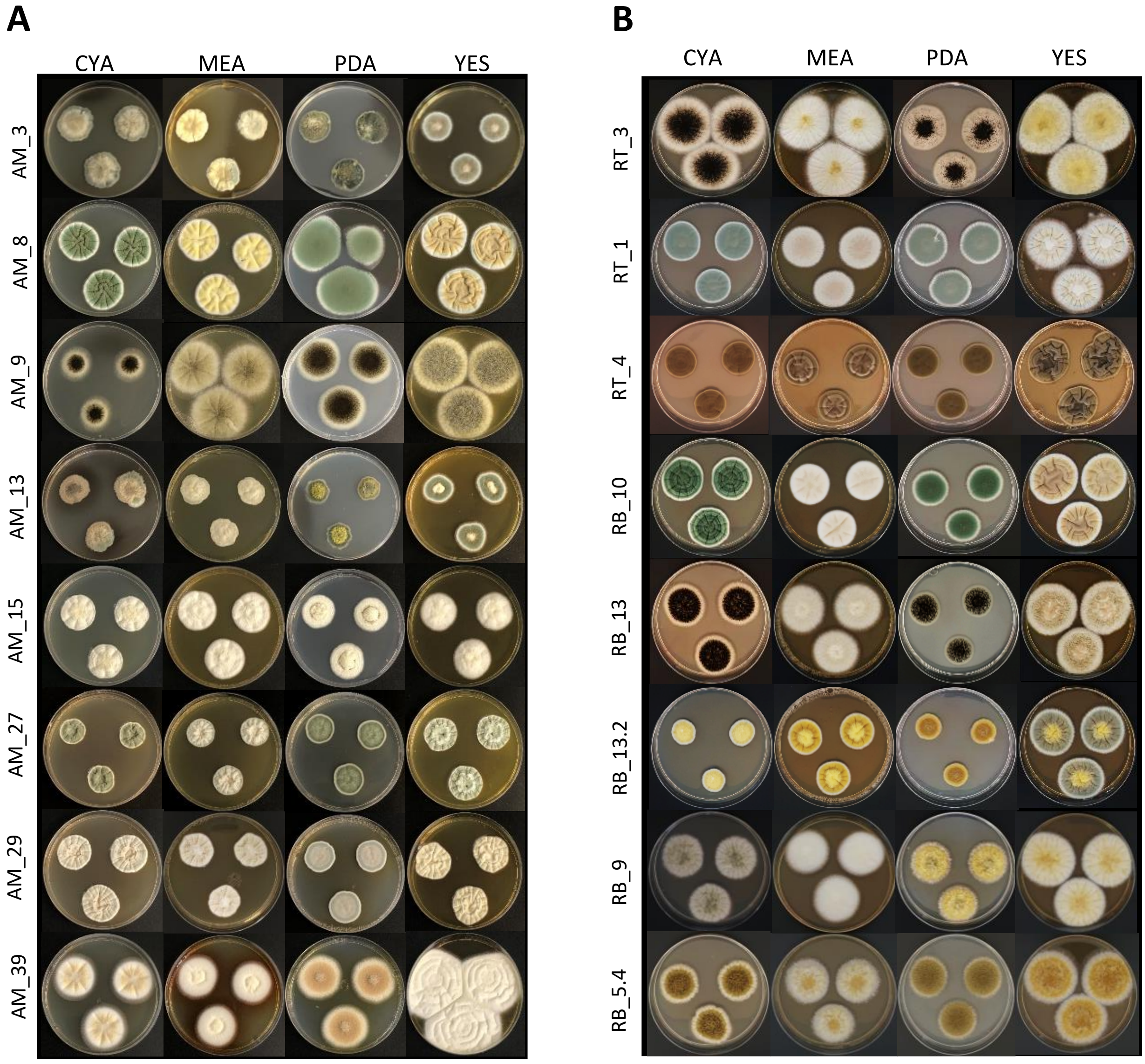

Supplementary Figure S1. Phenotypic characterization in CYA, MEA, PDA, and YES media of the different filamentous fungi isolated from rice bran from liquid (A) and solid (B) culture media.

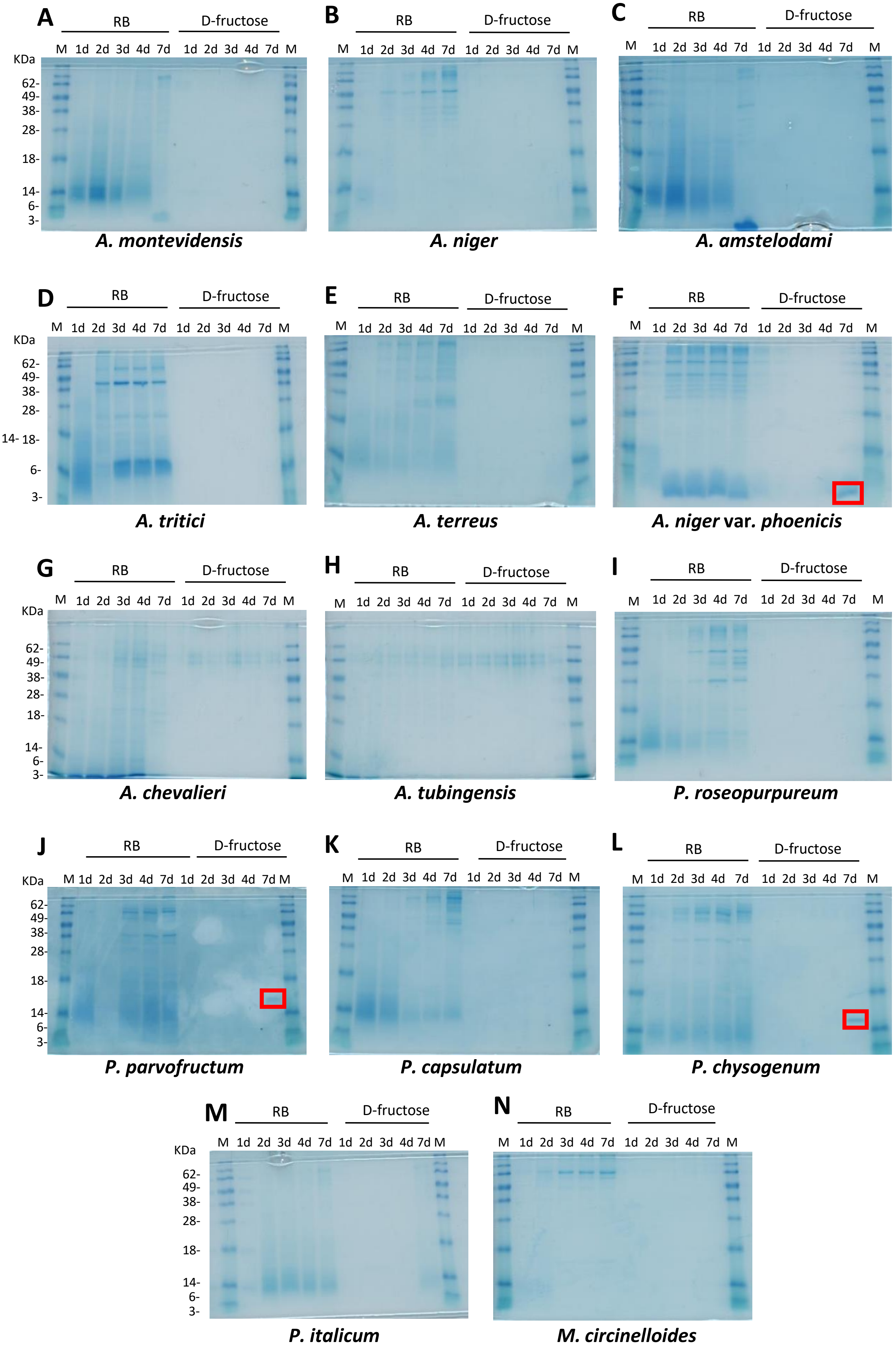

Supplementary Figure S2. SDS-PAGE analysis of the proteins secreted by *A. montevidensis* (A), *A. niger* (B), *A. amstelodami* (C), *A. tritici* (D), *A. terreus* (E), *A. niger* var. *phoenicis* (F), *A. chevalieri* (G), *A. tubingensis* (H), *P. roseopurpureum* (I), *P. parvofructum* (J), *P. capsulatum* (K), *P. chrysogenum* (L), *P. italicum* (M) and *M. circinelloides* (N) after 1, 2, 3, 4, and 7 days (d) of growth in rice bran (RB)-containing medium and D-fructose-containing medium. Bands selected for identification by proteomics are boxed.

A

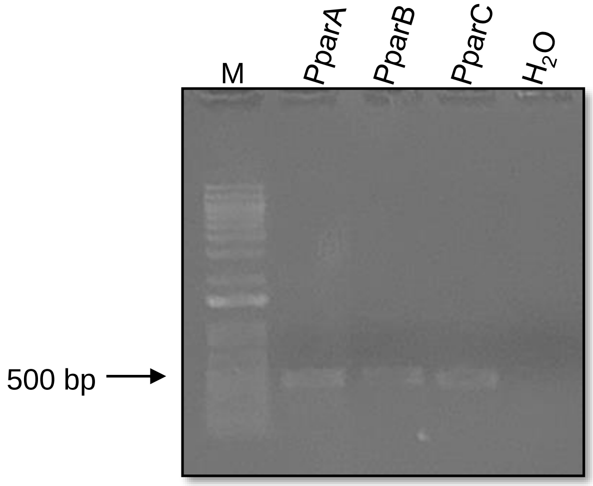

B

```
PAF           --ATGCAAATCACCACAGTTGCCCTTTTTCTCTTCGCTGCAATGGGCGGGGTAGCCACCC    58
PparA         TTATGCAAATCACCACAGTTGCCCTTTTTCTCTTCGCTGCAATGGGCGGGGTAGCCACCC    60
                **********************************************************

PAF           CCATTGAGTCTGTATCAAACGACCTCGATGCCAGGGCTGAGGCCGGTGTCCTGGCCAAAT    118
PparA         CCATTGAGTCTGTATCAAACGACCTCGATGCCAGGGCTGAGGCCGGTGTCCTGGCCAAAT    120
              ************************************************************

PAF           ACACCGGAGTGAGTAAACATCAATATCCCATACCTTAAGTACTCACTTGGGAATCGCGAC    178
PparA         ACACCGGAGTGAGTAAACATCAATATCCCATACCTTAAGTACTCACTTGGGAATCGCGAC    180
              ************************************************************

PAF           TAACGGTTCGGGACCACAACTCAGAAATGCACCAAATCTAAGAACGAATGTAAATACAAG    238
PparA         TAACGGTTCGGGACCACAACTCAGAAATGCACCAAATCTAAGAACGAATGTAAATACAAG    240
              ************************************************************

PAF           AACGATGCTGGAAAGGACACTTTTATCAAGTGCCCCAAGTTTGATAACAAGAAGGTAGAA    298
PparA         AACGATGCTGGAAAGGACACTTTTATCAAGTGCCCCAAGTTTGATAACAAGAAGGTAGAA    300
              ************************************************************

PAF           TATCAATCATTCGGAAGTAGCCATCTGAATCGATTTCGTGCTAATCTCGCTCTTTTTTCC    358
PparA         TATCAATCATTCGGAAGTAGCCATCTGAATCGATTTCGTGCTAATCTCGCTCTTTTTTCC    360
              ************************************************************

PAF           AGTGCACCAAGGATAATAACAAATGTACCGTCGACACCTACAACAACGCTGTCGATTGTG    418
PparA         AGTGCACCAAGGATAATAACAAATgTACCGTCGACACCTACAACAACGCTGTCGATTGTG    420
              ************************************************************

PAF           ACTAG   423
PparA         ACTAG   425
              *****
```

C

```
PcPAFB    -ATGCATATTACTAGCATTGCCATTGTCTTCTTCGCCGCAATGGGCGCGGTTGCTAGCC   59
PparB     TATGCAGATCACCAGCATTGCCATTGTCTTCTTCGCCGCAATGGGCGCGGTTGCTAGCC   60
           ***** ** ** *********************************************

PAFB      CCATCGCGACCGAGTCGGACGATCTTGATGCCCGAGACGTACAGCTTAGTAAATTCGGAG  119
PparB     CCATCGCGACCGAGTCGGACGATCTTGATGCCCGAGACGTACAGCTTAGTAAATTCGGAG  120
          ************************************************************

PAFB      GAGTAAGTTCTTCTTATAAGATGTCTATATAGAAATAGCACTAACCTTTCTGAACCGCTT  179
PparB     GAGTAAGTTCTTCTTATAAGATGTCTATGTGGAAATAGCACTAACCTTTCTGAACCACTT  180
          **************************** * ************************* ***

PAFB      TACAGGAATGCAGCTTGAAACACAACACGTGCACATACCTAAAGGGTGGAAAGAACCATG  299
PparB     CACAGGAATGCAGCTTGAAACACAACACGTGCACATACCTAAAGGGTGGAAAGAACCATG  300
           ***********************************************************

PAFB      TAGTCAATTGCGGTTCGGCCGCCAACAAGAAGGTAGATTCCGATTCGATTCGGGGCCAAT  359
PparB     TAGTCAATTGCGGTTCGGCCGCCAACAAGAAGGTAGATTCCGATTCGATTCGGGGCCAAT  360
          ************************************************************

PAFB      TGATTTGTTCTTATCATTTAATCTTCATCTACAGTGCAAGTCTGATCGCCACCACTGTG   419
PparB     TGATTTGTTCTTATCATTTAATCTTCATCTAAAGTGCAAGTCTGATCGCCACCACTGTG   420
          ******************************* ***************************

PAFB      AATACGATGAGCACCACAAGAGGGTTGACTGCCAGACCCCAGTTTGA     466
PparB     AATACGATGAGCACCACAAGAGGGTTGACTGCCAGACCCCAGTTTGA     467
          ***********************************************
```

D

```
PAFC      --ATGAAGGTTACTGCTCTCCTCTTCACCCTTATGGCTGCCACTGCCGTCAGTGCTTCAG   58
PparC     TAATGAAGGTTACTGCTCTCCTCTTCACCCTTATGGCTGCCACTGCCGTCAGTGCTTCAG   60
            **********************************************************

PAFC      TCTTGGATACACGAGACACCTGCGGAGGCGGCTATGGTGTTGATCAAAGACGTACAAACA   118
PparC     TCT-GGATACACGAGACACCTGCGGAGGCGGCTATGGTGTTGATCAAAGACGTACAAACA   119
          *** ********************************************************
PAFC      GCCCTTGCCAGGCCTCCAACGGAGACAGACACTTCTGCGGTTGTGACAGGACCGGTATTG   178
PparC     GCCCTTGCCAGGCCTCCAACGGAGACAGACACTTCTGCGGTTGTGACAGGACCGGTATTG   179
          ************************************************************

PAFC      TATGTTGATGTACCTAGCCTAAAATATCTGATCATATTTGCTAATATATATTCCGGTAAA   238
PparC     TATGTTCATGTACCTAGCCTAAAATATCTGGTCATATTTGCTAATATATATTCCTGTGAA   239
          ****** *********************** *********************** ** **

PAFC      AAGGTCGAGTGCAAAGGCGGAAAGTGGACAGAGATTCAAGATTGTGGCGGGGCTTCTTGC   298
PparC     AAGGTCGAGTGCAAAGGCGGAAAGTGGACAGAGATTCAAGATTGTGGCGGGgCTTCTTGC   299
          ************************************************************

PAFC      CGCGGTGTTAGCCAGGGGGGAGCCAGATGCTAG 331
PparC     CGCGGTGTTAGCCAGGGGGGAGCCAGATGCTAG 332
          *********************************
```

Supplementary Figure S3. Sequencing results and protein identification of *P. parvofructum* AFPs. (A) PCR results of the amplification of *P. parvofructum* class A AFP (PparA), class B AFP (PparB) and class C AFP (PparC) nucleotide sequences from *P. parvofructum* genomic DNA with primers listed in Supplementary Table S1. Nucleotide sequences obtained from Sanger sequencing were aligned against the nucleotide coding sequences of *P. chrysogenum* PAF (B), PAFB (C) and PAFC (D). Signal peptides + propeptides are highlighted in blue. Introns are highlighted in grey. Nucleotide disparities are highlighted in red.
